# Prevalence, Risk Factors, Disease-Related Knowledge, and Vaccination Attitudes and Behaviors for Long COVID Among French Civil Servants: Cross-Sectional Survey

**DOI:** 10.2196/83323

**Published:** 2025-12-05

**Authors:** Florence Carrouel, Virginie-Eve Lvovschi, Benjamin du Sartz de Vigneulles, Maryem Rhanoui, Roger Salamon, Michel Lamure, Corélie Salque, Romain Lan, Claude Dussart

**Affiliations:** 1 Laboratory "Health, Systemic, Process" (P2S), UR4129, University Claude Bernard Lyon 1, 11 rue Guillaume Paradin, Lyon, 69008, France, 33 0478785745; 2 Research on Healthcare Performance Laboratory (INSERM U1290), University Claude Bernard Lyon 1, Lyon, France; 3 Hospices Civils de Lyon, Lyon, France; 4 Institute of Public Health, Epidemiology and Development (ISPED), Inserm U1219, University of Bordeaux, Bordeaux, France; 5 Laboratory ADES, Aix Marseille University, CNRS, EFS, Marseille, France

**Keywords:** long COVID, health literacy, Knowledge, Attitudes, and Behaviors, KAB, COVID-19 vaccination, public health

## Abstract

**Background:**

Long COVID affects millions worldwide, straining health systems and workforce stability. This first nationwide survey among French civil servants combines epidemiological assessment with a Knowledge, Attitudes, and Behaviors approach. Long COVID remains a diagnostic and epidemiological challenge with evolving symptoms and uncertain categorization, particularly among self-suspected cases. Beyond prevalence and risk factors, understanding behavioral dimensions is essential to developing prevention strategies and maintaining workforce resilience.

**Objective:**

This study aimed to (1) assess the prevalence of long COVID among French civil servants; (2) identify associated sociodemographic, occupational, and health-related factors; (3) assess disease-related knowledge of long COVID and (4) examine attitudes and behaviors regarding COVID-19 vaccination.

**Methods:**

This cross-sectional survey was conducted in 2024 among active or retired civil servants in France. A Knowledge, Attitudes, and Behaviors–validated questionnaire, based on World Health Organization guidelines, was used. Responses were compared across 4 COVID-19 status groups (no COVID, COVID-19 without long COVID, diagnosed long COVID, and suspected long COVID). Statistical analyses included univariate tests and multivariable logistic regressions to identify factors associated with diagnosed or suspected long COVID.

**Results:**

Among 3962 eligible respondents, 61 (1.54%; 95% CI 1.20‐1.97) reported a formal diagnosis of long COVID and 241 (6.08%; 95% CI 5.38‐6.87) without diagnosis. Diagnosed long COVID was significantly associated with long-term sick leave (odds ratio [OR] 1.15, 95% CI 1.03‐6.28; *P*=.04) and long-term illness coverage (OR 0.72, 95% CI 0.27‐0.92; *P*=.03). Suspected long COVID was associated with being in a relationship (OR 1.65, 95% CI 1.08‐2.52; *P*=.02), widowed (OR 2.25, 95% CI 1.18‐4.31; *P*=.01), and uncertain (OR 1.90, 95% CI 1.32‐2.74; *P*<.001) or incomplete COVID-19 vaccination status (OR 1.67, 95% CI 1.16‐2.42; *P*=.01). Knowledge scores differed significantly across groups (ANOVA *F*_3,3476_=24.31, *P*<.001; *χ*²_6_=54.92, *P*<.001), with diagnosed cases showing the highest proportion of high knowledge (13/61, 21%) compared to 12.4% in the non-COVID group. Among 61 diagnosed cases, 36 (59%; 95% CI 46.4‐70.5) were vaccinated, 13 (21%; 95% CI 12.9‐33.2) intended to get vaccinated, and 12 (20%; 95% CI 11.6‐31.3) remained unvaccinated; among suspected cases, these proportions were 173 (71.8%; 95% CI 65.9‐77.1), 30 (12.4%; 95% CI 8.8‐17.3), and 38 (15.8%; 95% CI 11.6‐21.0), respectively.

**Conclusions:**

Unlike previous studies that examined the clinical or behavioral factors separately, this nationwide analysis linked epidemiological data with knowledge and vaccination behaviors. Among French civil servants, long COVID remains underdiagnosed, where absenteeism and sick leave threaten essential services. The study highlights disparities in disease-related knowledge, vaccination attitudes, and behaviors, underlining the importance of workplace health education and systematic screening. Vaccination is associated with lower odds of long COVID, reinforcing its preventive value. Thus, findings reveal organizational implications and support workplace-based prevention strategies integrating vaccination promotion, early detection, and health literacy to sustain the resilience of public services.

## Introduction

Long COVID, or post-COVID-19 syndrome, is estimated to affect approximately 65 million people worldwide, underscoring its substantial and growing burden on global public health [1]. The World Health Organization (WHO) defined it in 2021 as a condition affecting people with a history of probable or confirmed SARS-CoV-2 infection, developing at least 3 months after the onset of COVID-19, with symptoms persisting for at least 2 months and not explained by another diagnosis [2,3]. This definition remains widely used in recent clinical and epidemiological research and has been complemented by reviews describing long COVID as a systemic, multiorgan disease involving viral persistence, immune dysregulation, endothelial dysfunction, and microthrombosis [1,4,5].

Epidemiological estimates of the proportion of people suffering from long COVID vary widely. A 2025 meta-analysis of 429 studies estimated a pooled global prevalence of around 36% among COVID-positive individuals, with higher estimates at longer follow-up [6]. In contrast, the Organization for Economic Co-operation and Development (OECD) survey data from primary care (Patient-Reported Indicator Surveys program) suggested that approximately 7% of adults across participating countries reported persistent symptoms consistent with long COVID [7], while national surveillance by the US Centers for Disease Control and Prevention continues to track variable prevalence across demographics and states [8]. In France, a large-scale population study estimated that approximately 4% of adults present manifestations compatible with long COVID, depending on the case definition applied [9]. Despite these differences, the burden is indisputable, and prevalence estimates vary because of inconsistent definitions (clinical diagnosis vs self-reported symptoms), thresholds for symptom duration, study populations, and data collection methods.

Symptom profiles are dominated by chronic fatigue, dyspnea, musculoskeletal pain, and cognitive complaints such as “brain fog” [1,10]. These symptoms often impair quality of life, social functioning, and the ability to work. Beyond physical manifestations, long COVID is strongly associated with psychological sequelae. Meta-analyses and OECD reports published in 2023‐2024 indicate increased risks of anxiety, depression, and posttraumatic stress disorder in affected patients, as well as significant losses in quality-adjusted life years across OECD countries [1,10,11]. This corroborates earlier findings in European cohorts, where long COVID was associated with markedly higher odds of depression and anxiety compared to unaffected individuals [12].

The impact on work capacity and professional life represents another major challenge. Long COVID patients report a reduced ability to work (0.62 points; 0.30‐0.95) than those without Long COVID [13]. While 60.9% of patients with long COVID return to work, a significant number have to modify their tasks or work schedules [14]. In addition, patients with long COVID have difficulty maintaining a normal social and family life due to fatigue and cognitive impairment [15].

Beyond its health-related consequences, long COVID also exerts a significant societal and economic burden, particularly by reducing workforce availability and increasing public spending [16]. Estimates indicate that the direct and indirect costs of long COVID range from US $864 billion to US $1.04 trillion in OECD countries [11]. It also leads to higher health care use, with treatment expenses reaching up to $9000 per patient annually [16]. Its impact on work dynamics is particularly worrying. One survey found that 44% of people with long COVID were out of the labor force, while 51% worked fewer hours. These workforce reductions exacerbate existing labor shortages and contribute to inflation [16]. The extent of workforce disorganization [17] represents a major challenge not only for the private sector, but also for public services. In France, where the public service accounts for a substantial share of national employment, these disruptions may impact the continuity and efficiency of essential services. Moreover, the public sector offers a particularly relevant context for studying the impact of long COVID on employment, as it includes a wide range of socioprofessional categories and age groups, making it broadly representative of the working population.

Vaccination continues to play a dual role in this context. Systematic reviews and meta-analyses published between 2023 and 2025 consistently demonstrate that vaccination prior to infection is associated with a lower risk of developing long COVID [18-20]. Some studies also suggest that vaccination after infection may reduce the risk or severity of persistent symptoms [19]. Attitudes towards vaccination among people with long COVID remain complex. While some patients report hesitancy or heightened concerns, others show increased acceptance, especially when informed about vaccination as a preventive measure against long-term complications. Recent evidence also highlights that vicarious experience—knowing someone affected by long COVID—is associated with higher perceived risk of COVID-19 and more favorable beliefs about vaccine efficacy [21,22].

To date, most studies have investigated either prevalence and risk factors or knowledge and attitudes separately. However, combining these dimensions in a single survey provides a more comprehensive perspective: epidemiological estimates quantify the burden of long COVID, while knowledge and attitudes provide insight into the behavioral and organizational responses that influence prevention, early detection, and workforce resilience. This integrated approach is particularly relevant for civil servants, where both individual health and the continuity of essential public services are at stake.

Thus, this study aims to (1) assess the prevalence of long COVID among French civil servants; (2) identify associated sociodemographic, occupational, and health-related factors; (3) assess disease-related knowledge of long COVID; and (4) examine attitudes and behaviors regarding COVID-19 vaccination.

## Methods

### Study Design

This study was designed as a cross-sectional, anonymous questionnaire-based survey conducted between January 15, 2024, and February 12, 2024. It followed the CROSS (Checklist for Reporting of Survey Studies; Checklist 1) [23].

The study was supported by *Union Prévention Santé pour la Fonction publique* (UROPS), an entity operating under the supervision of public authorities, bringing together 11 mutual insurance organizations and overseeing the health coverage of approximately 1.7 million beneficiaries under the Morice Law of 1947 [24]. UROPS promotes preventive health care through the Compulsory Health Insurance Scheme. Regularly, it conducts the nationwide “Baromètre Santé” multisection survey to assess beneficiaries’ Knowledge, Attitudes, and Behaviors (KAB) on health issues, guiding targeted prevention programs. Findings from previous editions were published [25]. This study analyzes the long COVID section introduced in the 2024 edition.

### Study Population

The study population met the following inclusion criteria: (1) individuals aged ≥18 years, (2) active or retired civil servants, (3) affiliated with the UROPS insurance union for public sector workers, (4) registered on the public insurance platform [26] and having consented to be contacted, and (5) fluent in French; (6) willing to complete a questionnaire. Individuals were excluded if they were third-party beneficiaries of UROPS members or held nonpermanent employment status.

### Sample Size

Based on 95% CI, a margin of error of 5%, and an assumed response rate of 50%, a conservative estimate for unknown proportions, the minimum required sample size was calculated to be at least 384 participants. This calculation was made using the standard formula for sample size estimation in survey studies [27], considering the total number of public service persons listed by the UROPS (836,307 mailing or email addresses in the database).

### Sample Method

The population was recruited based on a convenience sampling methodology. Participants were recruited via a mail or email invitation sent by UROPS to its policyholders. UROPS covers approximately one-third of the State civil service workforce in France [24,28], including diverse occupational and regional categories. This framework provides access to a large and heterogeneous, although nonprobabilistic, sample of civil servants representative of multiple administrative branches.

### Development and Validation of the KAB Questionnaire

#### Questionnaire Design and Validation

A structured French questionnaire was developed to evaluate disease-related knowledge of long COVID and attitudes and behaviors regarding COVID-19 vaccination (Multimedia Appendix 1), following WHO methodological recommendations [29]. Two public health researchers conducted a literature review to identify existing KAB tools, from which items were selected, adapted, or newly formulated. The initial version included 39 closed-ended questions.

Validation followed the approach of Andrade et al [30], combining face and content validation.

In the first validation phase, a multidisciplinary panel of experts (2 general practitioners, 1 microbiologist, and 1 public health specialist) evaluated each item for clarity and relevance. Using a standardized form, experts rated each item as 2=satisfactory or 1=unsatisfactory. The Item-level Content Validity Index (I-CVI) was calculated as the proportion of experts rating the item as “relevant.” Following Polit and Beck [31], a minimum acceptable threshold of 0.75 (ie, at least 3 out of 4 experts in agreement) was applied: items with an I-CVI of ≥0.75 were retained, while those below this threshold were revised or removed. The Scale-level Content Validity Index (S-CVI/Ave) was then calculated as the average of the I-CVI across all items. The updated version was re-evaluated and approved by the expert panel. The second phase involved cognitive debriefing with 6 individuals not included in the main study. They reviewed the questionnaire and provided feedback on clarity and comprehension, leading to minor wording refinements.

The expert committee then validated this final version, which comprised 36 closed-ended questions: 12 questions on sociodemographic characteristics (Q1-Q12), 5 questions on health status (Q13-Q17), 10 questions to assess whether participants had been diagnosed with or likely experienced long COVID (Q18-Q27), 7 questions on long COVID knowledge (Q28-Q34), and 2 questions on attitudes and behavior regarding COVID-19 vaccination (Q35 and Q36), which explored participants’ intentions to vaccinate and their motivations, providing information on at least 7 specific behaviors or attitudes via Q36.

#### Construction of the Knowledge Score and Analysis of Attitude and Behavior Answers

A scoring system was developed to assess respondents’ knowledge of long COVID. From Q28 to Q34 (long COVID knowledge), a total of 9 correct answers distributed across the relevant items. Correct and incorrect answers were determined based on information published by the French High Health Authority (*Haute Autorité de la Santé* [HAS]) [32]. A binary scoring method was applied, assigning +1 point for each correct answer and 0 for incorrect ones. The resulting knowledge score ranged from 0 to 9 (9 items) and was categorized as follows: 0-3 (low), 4-6 (medium), and 7-9 (high).

For attitudes and behaviors related to COVID-19 vaccination, a nonscoring analysis was conducted.

#### Survey Administration

Participants could complete the paper questionnaire or the online questionnaire. For the paper questionnaire, respondents had to return it using a prepaid envelope. For the online questionnaire, the “Confirmit” software (SurveySparrow) with anonymized responses was used, and to prevent multiple submissions, people contacted by email had an individual weblink that could only be used once. Participants could also pause and resume the questionnaire if needed. After validation, the web link was no longer accessible.

### Definition of COVID-19 Status Groups

Long COVID was defined according to the HAS recommendations as the persistence of one or more symptoms beyond 4 weeks after acute infection, without another diagnosis explaining them [33]. This operational threshold is shorter than the WHO definition, which requires symptoms to persist for at least 12 weeks [34]. Therefore, caution is needed when comparing our results with international studies based on the WHO definition.

Participants were categorized into four mutually exclusive groups based on several answers to questions (Multimedia Appendix 2):

1. No COVID: never infected with SARS-CoV-2.
2. COVID-19 without long COVID: previous infection without persistent symptoms compatible with long COVID.
3. Diagnosed long COVID: formal medical diagnosis of long COVID following confirmed SARS-CoV-2 infection.
4. Suspected long COVID: persistent symptoms consistent with long COVID but without a formal medical diagnosis.

This approach distinguishes between medically confirmed cases and self-reported symptom persistence, but it also carries a risk of misclassification: undiagnosed individuals may underestimate their condition, while some self-reported cases could include symptoms attributable to other causes.

### Statistical Analysis

Statistical analyses were performed using Python (version 3.10; Python Software Foundation) with *pandas*, *numpy*, and *statsmodels* libraries. Descriptive statistics summarized variables including gender, age, marital and parental status, region, employment, statutory category, education, chronic conditions, and administration type. Frequencies and percentages were used for categorical variables; medians and IQR for continuous and ordinal variables. Univariate analyses compared the 4 COVID-19 groups (no infection, infection without long COVID, suspected long COVID, and diagnosed long COVID) using chi-square test for categorical variables and Kruskal-Wallis or Mann-Whitney *U* tests for ordinal or continuous variables. The Spearman correlation assessed ordinal associations.

Multivariable logistic regressions identified factors associated with diagnosed or suspected long COVID. Covariates for multivariable logistic models were selected a priori based on clinical and epidemiological plausibility (age, gender, education, chronic disease, long-term illnesses coverage, vaccination status, and knowledge score K) and were not removed solely on the basis of univariate significance. All variables corresponded directly to questionnaire items, and no post hoc transformations or recoding were performed. Variables with *P*<.1 in univariate screening or strong subject-matter justification were retained. Prespecified interactions (chronic condition [Q13] × vaccination status [Q35], age [Q2] × gender [Q1], education [Q8] × score K, and group × score K) were assessed via likelihood-ratio tests; none met retention criteria, and final results are reported for main effects models. Results were expressed as odds ratios (OR) with 95% CI, significance set at *P*<.05.

No missing data were present in the final analytical dataset: the questionnaires retained for analysis were fully completed. Consequently, no imputation or missing data mechanism analyses (eg, missing completely at random tests) were required. As no missing data or imputed variables were involved, no sensitivity analyses were necessary. Similarly, no weighting procedures were applied, as analyses were conducted on the complete dataset of eligible respondents. No specific analysis of nonresponse bias was performed, as only aggregate demographic data of the target population were available.

The exposures included sociodemographic, occupational, and health-related characteristics, as well as COVID-19 vaccination status, while the outcomes were diagnosed long COVID and suspected long COVID. KAB responses were compared across groups: knowledge was analyzed with Kruskal-Wallis tests, and attitudes and behaviors were described but not modeled.

### Ethical Considerations

UROPS regularly conducts the *Baromètre Santé* survey [25]. The research team designed a specific KAB module on long COVID, which was incorporated into the 2024 edition. As the survey generated fully anonymized data, no prior approval was required for its administration. Nevertheless, the research protocol was submitted to the Aix Marseille University ethics committee, which granted approval on July 17, 2025 (n° 2025-07-17-02), in accordance with national procedures for the secondary use of anonymized survey data. This study followed the Declaration of Helsinki and the reference methodology MR-004 of the French National Commission on Information Technology and Liberties. All participants provided informed consent prior to completing the questionnaire. Consent was obtained electronically for online respondents and in written form for postal respondents. Participation was voluntary, responses were anonymous, and data were used exclusively for research purposes. To ensure confidentiality, responses were completely anonymous at the time of collection and no personal identifiers were recorded. For the online questionnaire, access was provided via secure individual links that could only be used once, and returned paper questionnaires contained no identifying information. Participants did not receive any financial or nonfinancial compensation for their participation. No images or supplementary materials that could allow the identification of individual participants are included in this paper.

## Results

### Psychometric Properties of the Questionnaire

Content validity was assessed in two rounds. In the first round, 39 items were evaluated by 4 experts. I-CVI values ranged from 0.50 to 1.00, and the S-CVI/Ave was 0.87. Three items with I-CVI <0.75 were eliminated at this stage. In the second round, the revised questionnaire (36 items) was re-evaluated by the same panel. Final indices indicated strong agreement, with I-CVI values ranging from 0.75 to 1.00 and an S-CVI/Ave of 0.92, confirming satisfactory content validity.

### Sociodemographic and Health Characteristics of Respondents

**Figure 1.**
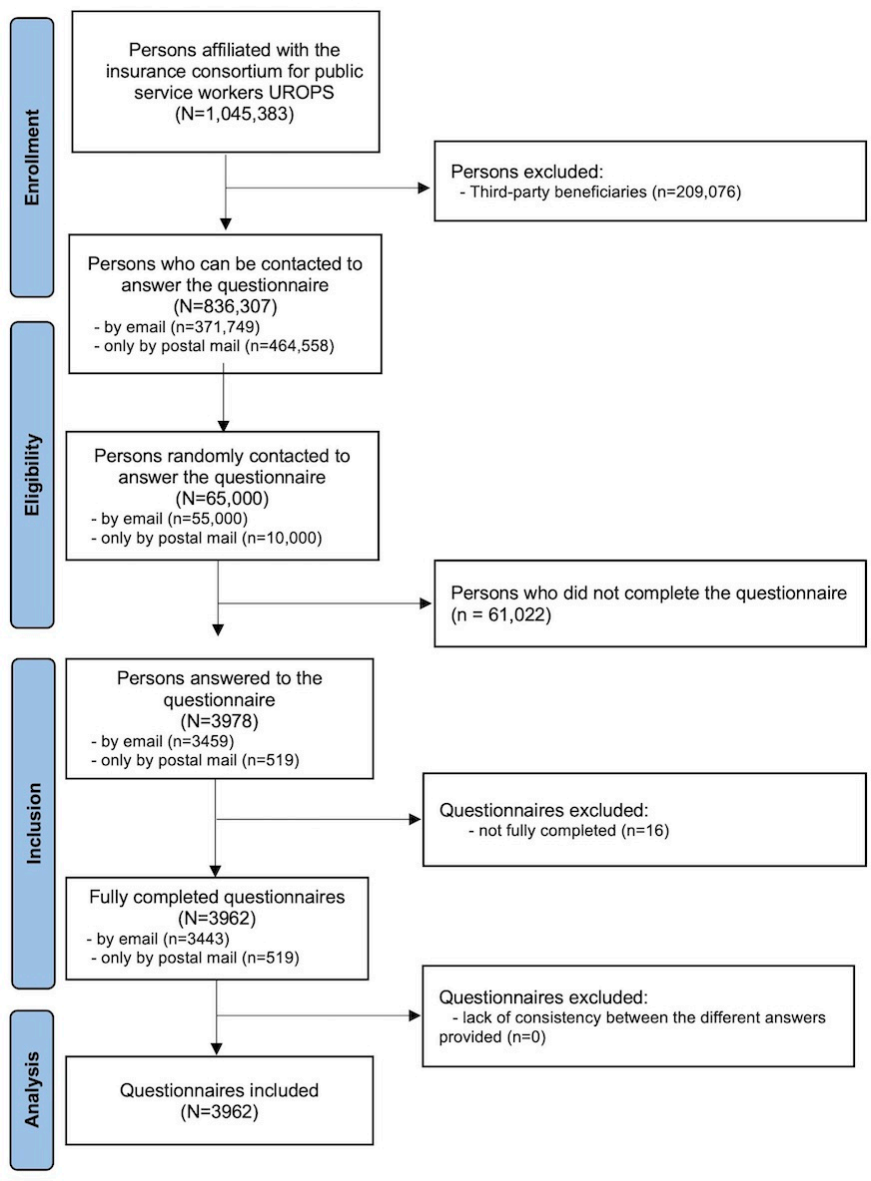
Flowchart of the study. UROPS: Union Prévention Santé Pour La Fonction Publique.

Among the 1,045,383 individuals affiliated with UROPS in 2024, 209,076 were third-party beneficiaries (dependents of primary insured members) who could not be contacted directly because their consent for communication was not authorized. Among the remaining 836,307 eligible policyholders, 65,000 were invited to participate. 3978 (6.12%) insured participated in this survey. 3962 questionnaires were considered eligible, with all questions answered, whereas 16 were excluded because participants dropped out during the survey (Figure 1). No information was available on reasons for nonparticipation, as data collection was fully anonymized and participation was voluntary.

Among the 3962 respondents, 1091 (27.54%) reported never having had a COVID-19 infection, 2087 (52.68%) indicated a previous infection without subsequent long COVID symptoms, 61 (1.54%) had a confirmed diagnosis of long COVID, and 241 (6.08%) reported persistent symptoms consistent with long COVID despite not having received a formal medical diagnosis.

Sociodemographic, health, and occupational characteristics were described in Table 1 and Multimedia Appendix 3. Compared with national statistics on the French civil service [28], the sample displayed a broadly similar gender and age distribution. However, category B agents were slightly overrepresented (1466/3962, 37.00% vs 616,800/2,570,000, 24.00%) and category A agents underrepresented (1652/3962, 41.70% vs 1,440,000/2,570,000, 56.00%), suggesting moderate structural differences but overall demographic consistency.

**Table 1.** Sociodemographic and health characteristics of the respondents.

Variable	All, n (%; 95% CI)	No COVID-19^a^, n (**%**; 95% CI)	COVID-19 without long COVID^b^, n (**%**; 95% CI)	Diagnosed long COVID^c^, n (**%**; 95% CI)	Suspected long COVID^d^, n (**%**; 95% CI)
Gender					
Male	1823 (46.01; 44.46‐47.56)	659 (47.8 ;45.2‐50.5)	959 (46.0; 43.8‐48.1)	22 (36; 24‐48)	96 (39.8; 33.7‐46.0)
Female	2139 (53.99; 52.44‐55.54)	719 (52.2; 49.5‐54.8)	1128 (54.0; 51.9‐56.2)	39 (64; 52‐76)	145 (60.2; 54.0‐66.3
Age (y)					
18‐29	27 (0.68; 0.43‐0.94)	7 (0.5; 0.1‐0.9)	14 (0.7; 0.3‐1.0)	0 (0; 0‐0)	3 (1.2; 0.0‐2.6)
30‐39	179 (4.52; 3.87‐5.16)	40 (2.9; 2.0‐3.8)	106 (5.1; 4.1‐6.0)	7 (12; 4‐20)	14 (5.8; 2.9‐8.8)
40‐49	512 (12.92; 11.88‐13.97)	111 (8.1; 6.6‐9.5)	333 (16.0; 14.4‐17.5)	13 (21; 11‐32)	27 (11.2; 7.2‐15.2)
50‐59	875 (22.08; 20.79‐23.38)	252 (18.3; 16.2‐20.3)	486 (23.3; 21.5‐25.1)	17 (28; 17‐39)	77 (32.0; 26.1‐37.8)
60‐69	1250 (31.55; 30.10‐33.00)	458 (33.2; 30.7‐35.7)	657 (31.5; 29.5‐33.5)	16 (26; 15‐37)	69 (28.6; 22.9‐34.3)
70‐79	974 (24.58; 23.24‐25.92)	437 (31.7; 29.3‐34.2)	436 (20.9; 19.1‐22.6)	7 (12; 4‐20)	45 (18.7; 13.8‐23.6)
≥80	145 (3.66; 3.08‐4.24)	73 (5.3; 4.1‐6.5)	55 (2.6; 1.9‐3.3)	1 (2; 0‐5)	6 (2.5; 0.5‐4.5)
Marital status					
Single	569 (14.36; 13.27‐15.45)	232 (16.8; 14.9‐18.8)	273 (13.1; 11.6‐14.5)	16 (26; 15‐37)	28 (11.6; 7.6‐15.7)
Couple	2646 (66.78; 65.32‐68.25)	842 (61.1; 58.5‐63.7)	1477 (70.8; 68.8‐72.7)	31 (51; 38‐63)	168 (69.7; 63.9‐75.5)
Divorced	495 (12.49; 11.46‐13.52)	181 (13.1; 11.4‐14.9)	243 (11.6; 10.3‐13.0)	12 (20; 10‐30)	26 (10.8; 6.9‐14.7)
Widowed	252 (6.36; 5.60‐7.12)	123 (8.9; 7.4‐10.4)	94 (4.5; 3.6‐5.4)	2 (3; 0‐8)	19 (7.9; 4.5‐11.3)
Current employment status					
Working	1724 (43.51; 41.97‐45.06)	452 (32.8; 30.3‐35.3)	1020 (48.9; 46.7‐51.0)	36 (59; 47‐71)	127 (52.7; 46.4‐59.0)
Parental leave	4 (0.10; 0.00‐0.20)	0 (0; 0.0‐0.0)	4 (0.2; 0.0‐0.4)	0 (0; 0‐0)	0 (0.0; 0.0‐0.0)
In school, training, and internship	11 (0.28; 0.11‐0.44)	6 (0.4; 0.1‐0.8)	4 (0.2; 0.0‐0.4)	0 (0; 0‐0)	1 (0.4; 0.0‐1.2)
Retired	2118 (53.46; 51.90‐55.01)	883 (64.1; 61.5‐66.6)	1014 (48.6; 46.4‐50.7)	18 (30; 18‐41)	107 (44.4; 38.1‐50.7)
On disability	16 (0.40; 0.21‐0.60)	10 (0.7; 0.3‐1.2)	4 (0.2; 0.0‐0.4)	0 (0; 0‐0)	1 (0.4; 0.0‐1.2)
On sick leave for >3 months	89 (2.25; 1.78‐2.71)	27 (2; 1.2‐2.7)	41 (2; 1.4‐2.6)	7 (12; 4‐20)	5 (2.1; 0.3‐3.9)
Last statutory category					
Category A+	661 (16.68; 15.52‐17.84)	221 (16; 14.1‐18.0)	390 (18.7; 17.0‐20.4)	5 (8; 1‐15)	31 (12.9; 8.6‐17.1)
Category A	991 (25.01; 23.66‐26.36)	318 (23.1; 20.9‐25.3)	575 (27.6; 25.6‐29.5)	16 (26; 15‐37)	55 (22.8; 17.5‐28.1)
Category B	1466 (37.00; 35.50‐38.50)	511 (37.1; 34.5‐39.6)	754 (36.1; 34.1‐38.2)	26 (43; 30‐55)	95 (39.4; 33.2‐45.6)
Category C	685 (17.29; 16.11‐18.47)	263 (19.1; 17.0‐21.2)	297 (14.2; 12.7‐15.7)	13 (21; 11‐32)	47 (19.5; 14.5‐24.5)
Contract	21 (0.53; 0.30‐0.76)	7 (0.5; 0.1‐0.9)	10 (0.5; 0.2‐0.8)	0 (0; 0‐0)	3 (1.2; 0.0‐2.6)
Other	138 (3.48; 2.91‐4.05)	58 (4.2; 3.1‐5.3)	61 (2.9; 2.2‐3.6)	1 (2; 0‐5)	10 (4.1; 1.6‐6.7)
Level of study					
Level I^e^	994 (25.09; 23.74‐26.44)	401 (29.1; 26.7‐31.5)	435 (20.8; 19.1‐22.6)	15 (25; 14‐35)	68 (28.2; 22.5‐33.9)
Level II^f^	1596 (40.28; 38.76‐41.81)	532 (38.6; 36.0‐41.2)	855 (41; 38.9‐43.1)	31 (51; 38‐63)	94 (39.0; 32.8‐45.2)
Level III^g^	1372 (34.63; 33.15‐36.11)	445 (32.3; 29.8‐34.8)	797 (38.2; 36.1‐40.3)	15 (25; 14‐35)	79 (32.8; 26.9‐38.7)
Chronic disease or disability or health problem					
Yes	1840 (46.44; 44.89‐47.99)	637 (46.2; 43.6‐48.9)	958 (45.9; 43.8‐48.0)	39 (64; 52‐76)	107 (44.4; 38.1‐50.7)
No	1976 (49.87; 48.32‐51.43)	690 (50.1; 47.4‐52.7)	1069 (51.2; 49.1‐53.4)	19 (31; 20‐43)	120 (49.8; 43.5‐56.1)
Do not want to answer	0 (0.00; 0.00‐0.00)	0 (0; 0.0‐0.0)	0 (0; 0.0‐0.0)	0 (0; 0‐0)	0 (0.0; 0.0‐0.0)
Long-term illnesses coverage^h^					
Yes	1175 (29.66; 28.23‐31.08)	461 (33.5; 31.0‐35.9)	553 (26.5; 24.6‐28.4)	28 (46; 33‐58)	63 (26.1; 20.6‐31.7)
No	2706 (68.30; 66.85‐69.75)	892 (64.7; 62.2‐67.3)	1493 (71.5; 69.6‐73.5)	31 (51; 38‐63)	175 (72.6; 67.0‐78.2)
Do not know	0 (0.00; 0.00‐0.00)	0 (0; 0.0‐0.0)	0 (0; 0.0‐0.0)	0 (0; 0‐0)	0 (0.0; 0.0‐0.0)

a Participants who reported never having had a SARS-CoV-2 infection.

b Participants who reported a previous SARS-CoV-2 infection but no persistent symptoms compatible with long COVID.

c Participants who reported a formal medical diagnosis of long COVID following a confirmed SARS-CoV-2 infection.

d Participants who reported at least one persistent symptom consistent with long COVID beyond four weeks after the acute phase of infection but without a formal medical diagnosis.

e Level of study < “baccalaureate”.

f Level of study ≥ “baccalaureate” degree and ≤ “baccalaureate” degree +2 years.

g Level of study > “baccalaureate” degree >2 years.

h Reimbursed at 100% by the health insurance for a long-term illness.

### Determinants of Diagnosed or Suspected Long COVID Status in French Civil Servants

**Figure 2.**
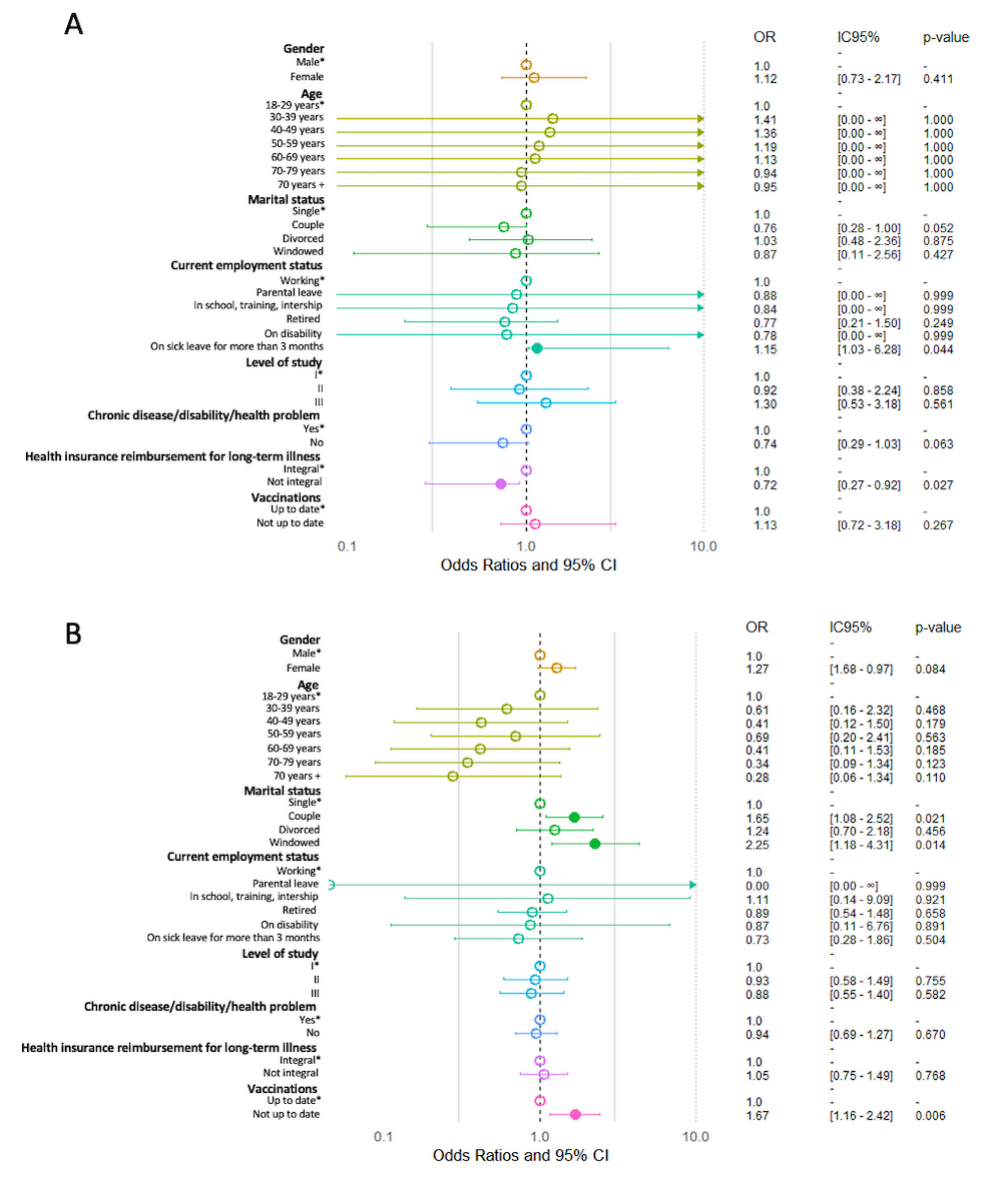
Binary logistic regression performed to evaluate the determinants of (A) diagnosed or (B) suspected long COVID. Odds ratios are presented as points, with horizontal error bars representing 95% CIs. Level of study I: below baccalaureate degree; level of study II: greater than or equal to baccalaureate degree and less than or equal to baccalaureate degree plus 2 years; and level of study III: greater than or equal to baccalaureate degree plus 2 years.

The results of the binary logistic regressions performed to evaluate the determinants diagnosed long COVID and suspected long COVID are presented in Figure 2.

In the multivariable models (Figure 2), diagnosed long COVID was significantly associated with employment-related variables. Participants on sick leave for >3 months had higher odds of receiving a diagnosis (OR 1.15, 1.03‐6.28; *P*=.04), while those without full long-term illness reimbursement had lower odds (OR 0.72, 95% CI 0.27‐0.92; *P*=.03). No associations were found with age, gender, education, or chronic conditions. For suspected long COVID, marital status and vaccination status remained significant. Compared to single individuals, those who were in a relationship (OR 1.65, 95% CI 1.08‐2.52; *P*=.02) and widowed participants (OR 2.25, 95% CI 1.18‐4.31; *P*=.01) had higher odds of reporting symptoms. Furthermore, being not up to date with COVID-19 vaccination (OR 1.67, 95% CI 1.16‐2.42; *P*=.01) or uncertain about vaccination status (OR 1.90, 95% CI 1.32‐2.74; *P*<.001) was also significantly associated with suspected long COVID. Other covariates, including age, gender, occupational status, and long-term illness coverage, were not significant.

### Analysis of KAB Regarding Long COVID Disease and Vaccination

#### Knowledge Score Related to Long COVID Among French Civil Servants

**Figure 3.**
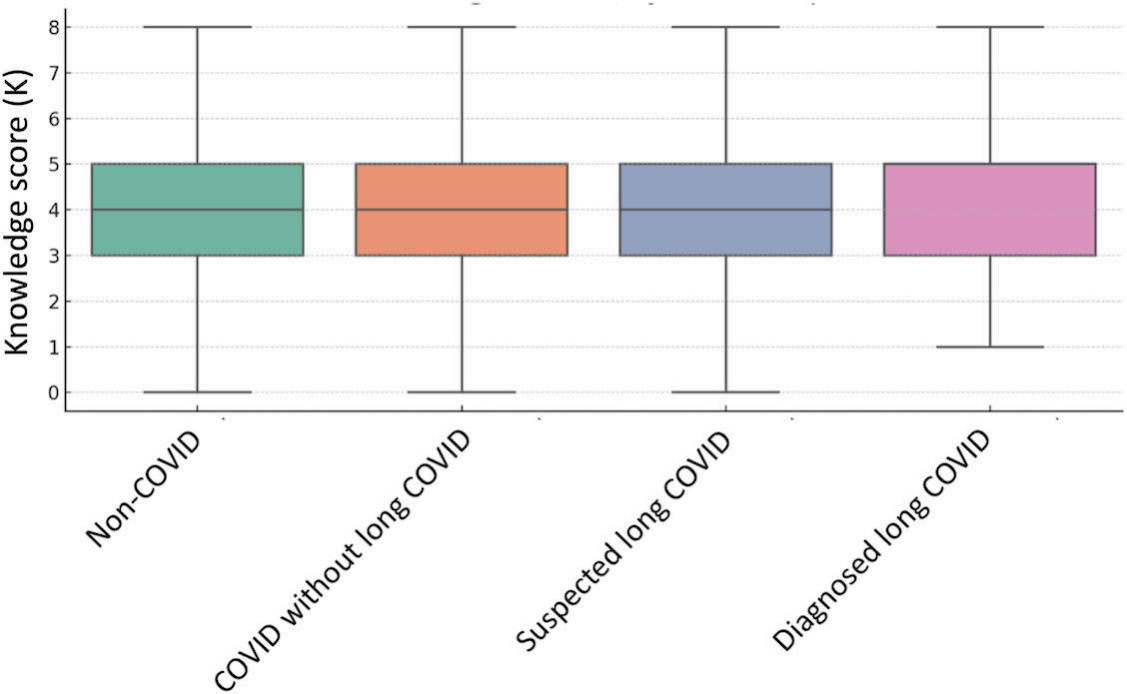
Knowledge score regarding long COVID among French civil servants.

Knowledge scores varied according to participants’ COVID-19 status (Figure 3). The diagnosed long COVID group reported the highest median score of 5.0 (IQR 3.0-5.0), followed by the suspected long COVID, COVID without long COVID, and non-COVID groups (all with a median 4.0, IQR 3.0-5.0 score). Despite similar medians across most groups, the distribution of scores differed.

The Kruskal-Wallis test indicated a statistically significant difference in score distribution between groups (H=82.55; *P*<.001), suggesting that knowledge levels are associated with prior COVID-19 exposure and long COVID diagnosis. The diagnosed long COVID group had the highest proportion of high knowledge scores (21.3%; 95% CI 12.9‐33.1), while the non-COVID groups showed a higher rate of low scores (23.6%; 95%CI 21.1‐26.2; Multimedia Appendix 4). Post hoc chi-square pairwise comparisons showed that individuals in the non-COVID group had significantly lower knowledge scores compared to all other groups, including those with diagnosed long COVID (mean difference –0.72; *P*<.001), suspected long COVID (mean difference –0.35; *P*<.001), and COVID without long COVID (mean difference –0.47; *P*<.001).

#### Attitudes and Behavior Regarding Vaccination According to Long COVID Status

Attitudes and behaviors regarding vaccination diagnosed or suspected population with long COVID are presented in Table 2. A total of 13 (21%; 95%CI 13‐33) respondents who intended to vaccinate against COVID-19 had been diagnosed with long COVID, while 30 (12.4%; 95% CI 8.8‐17.3) reported suspected long COVID symptoms. In contrast, a significantly higher proportion of respondents who declared they were already vaccinated reported a history of long COVID, with 36 (59.0%; 95% CI 46.4‐70.5) diagnosed and 173 (71.8%; 95% CI 65.9‐77.1) suspected cases. Finally, among those who did not intend to get vaccinated and were not yet vaccinated, 12 (20%; 95% CI 12‐31) had diagnosed long COVID and 38 (15.8%; 95% CI 11.6‐21.0) had suspected long COVID.

**Table 2.** Analysis of attitudes and behavior related to COVID-19 vaccination among diagnosed (n=61) and suspected (n=241) population with long COVID.

	Diagnosed long COVID, n (%; 95% CI)	Suspected long COVID, n (%; 95% CI)
Do you intend to vaccinate against COVID-19?		
Yes	13 (21; 13‐33)	30 (12.4; 8.8‐17.3)
No, I’m already vaccinated	36 (59; 46‐70)	173 (71.8; 65.9‐77.1)
No, and I’m not vaccinated	12 (20; 12‐31)	38 (15.8; 11.6‐21.0)
For what reason(s) do you not intend to be vaccinated against COVID-19?		
Not effective in preventing me from getting sick	5 (19; 8‐37)	8 (18.2; 9.5‐32.1)
No confidence in vaccination, risk of side effects	4 (15; 6‐33)	9 (21.6; 11.9‐36.7)
No confidence in vaccine efficacy	4 (15; 6‐33)	6 (14.8; 6.9‐28.8)
I’m immune, already had COVID-19	2 (7; 2‐23)	4 (9.1; 3.6‐21.3)
It doesn’t matter if I’m infected by this virus, the disease is not serious	1 (4; 1‐18)	3 (6.8; 2.4‐18.7)
Prefer other means of prevention, such as barrier gestures, wearing a mask or physical distancing	6 (22; 11‐42)	6 (13.6; 5.9‐28.0)
I don’t like shots	1 (4; 1‐18)	1 (2.3; 0.4‐11.8)
Against vaccination in general	0 (0; 0-0)	1 (2.3; 0.4‐11.8)
Too complicated to get vaccinated	0 (0; 0-0)	1 (2.3; 0.4‐11.8)
There is currently little or no COVID-19	0 (0; 0-0)	3 (6.8; 2.4‐18.7)
Other reasons	4 (15; 6‐33)	2 (4.6;1.3‐15.3)
For what reason(s) do you intend to be vaccinated against COVID-19?		
The COVID-19 vaccine has already protected me from a more serious form.	6 (16; 7‐33)	10 (12.5; 6.9‐21.6)
I need to protect myself from new variants	5 (14; 6‐28)	16 (20; 12.7‐30.1)
I need to boost my immunity	7 (19; 9‐35)	14 (17.5; 10.7‐27.4)
I need to protect my loved ones	9 (24; 14‐40)	17 (21.3; 13.7‐31.8)
I must follow the advice of my doctor/pharmacist.	7 (19; 9‐35)	13 (16.3; 9.7‐26.1)
I follow the recommendations of health authorities	2 (5; 2‐18)	9 (11.3; 6.0‐20.3)
Other reason	1 (3; 1‐14)	1 (1.3; 0.2‐6.9)

## Discussion

### Principal Results and Comparison With Prior Work

To our knowledge, this study is the first to analyze the prevalence and characteristics of long COVID among French civil servants. It explores the associations between long COVID status and sociodemographic, occupational, and behavioral factors, along with long COVID knowledge, attitudes, and behaviors related to COVID-19 vaccination.

This study differs from prior research by combining epidemiological analysis with a validated KAB approach in a large population of French civil servants—a group critical to maintaining public service continuity, but previously overlooked in long COVID research. This dual perspective highlights not only medical determinants but also behavioral and organizational dimensions essential for prevention and resilience strategies.

The findings highlight significant diagnostic disparities, knowledge gaps, attitudes, and behavioral trends, while also suggesting that long COVID may interfere with functioning and professional stability, even though the quality of life was not directly assessed.

Among 3962 respondents, 2087 (52.68%) indicated a COVID-19 infection without subsequent long COVID symptoms, 61 (1.54%) had a confirmed diagnosis of long COVID, and 241 (6.08%) reported persistent symptoms consistent with long COVID despite not having received a formal medical diagnosis. These results are consistent with French estimates: of the 48% of individuals who had been infected with SARS-CoV-2 for >3 months, 8% met the criteria for post-COVID-19 condition [35], and fall within the ranges reported by the WHO (10%‐20%), the US Centers for Disease Control and Prevention (20%‐25%) [36], and international meta-analyses [37]. The high proportion of suspected undiagnosed cases highlights a possible underdetection, probably linked to barriers in access to care, poor recognition of symptoms, or reluctance to seek medical advice [38]. This underlines the importance of improving referral and screening in primary care.

Long COVID has a functional impact with a decline in well-being. Although the quality of life was not assessed through standardized tools (eg, 36-Item Short Form Health Survey and EuroQol 5 Dimensions), several questionnaire items can be interpreted as both risk factors and consequences for daily functioning. Participants with diagnosed long COVID were more often on long-term sick leave (11.5% vs 2.2% in the general population and 2% in the population of no long COVID), reflecting either preexisting vulnerability or reduced work capacity due to long COVID. Chronic conditions were more frequent among long COVID cases (46%), suggesting that underlying health status could influence both susceptibility and functional outcomes. These findings are consistent with previous studies indicating that long COVID reduces work capacity by 0.62 points on average [13], with 60% of patients needing workstation or schedule adaptations [39]. Mental health repercussions are also reported, with a higher prevalence of depression (OR 2.35, 95% CI 1.49‐3.70) and anxiety (OR 2.53, 95% CI 1.76‐3.61) [12]. In our study, respondents with diagnosed long COVID reported more comorbidities (respiratory diseases, musculoskeletal disorders, migraines, and mental health disorders such as depression, anxiety, or sleep disorders), reinforcing the hypothesis of multidimensional consequences [40].

Although mental health outcomes were not directly assessed in this study, the high prevalence of reported comorbidities such as depression, anxiety, and sleep disorders underscores the psychological burden of long COVID, which is well documented in the literature. Meta-analyses have shown increased risks of anxiety, depression, and cognitive impairment among affected individuals [41-43]. These manifestations can reduce productivity and slow return-to-work trajectories. These findings highlight the need to integrate mental health assessment and support into occupational reintegration programs in accordance with WHO recommendations for post-COVID management [44].

This study also supports evidence that vaccination is associated with lower odds of long COVID. While diagnosed or suspected cases were more frequent among vaccinated participants at the time of the survey (n=36, 59%; 46.4‐70.5 and n=173, 71.8%; 65.9‐77.1, respectively), this likely reflects postinfection vaccination, greater medical engagement, or higher health awareness, rather than causality. Conversely, unvaccinated individuals or those uncertain of their vaccination status had higher odds ratios of suspected long COVID (OR 1.67, 95% CI 1.16‐2.41; *P*=.006 and OR 1.90, 95% CI 1.32‐2.74; *P*<.001 respectively), aligning with studies reporting reduced incidence of long COVID after full vaccination [45,46].

The study reveals disparities in knowledge and health attitudes and behavior regarding long COVID. Knowledge was significantly higher among individuals who had experienced COVID-19, particularly those with a confirmed diagnosis, supporting evidence that personal illness experience enhances health-related engagement and information seeking [47,48]. In contrast, individuals who had never contracted COVID-19 scored lower, likely due to lower exposure and reduced perceived relevance [49]. Although knowledge scores differed significantly across groups, the absolute differences were small. Similar results were found in recent knowledge, attitude, and practice studies on long COVID and vaccination. He et al [50] found that modest knowledge score differences were nonetheless associated with greater adherence to preventive behaviors and vaccine acceptance, suggesting that even small awareness gaps may have behavioral implications. Lazarus et al [51] also showed that limited variations in COVID-19 knowledge and attitudes influenced vaccine perceptions and willingness to vaccinate. These results suggest that improving health literacy and risk perception remains essential. Strengthening disease literacy among unaffected populations is therefore crucial to encourage early recognition, care-seeking, and risk awareness. Workplace health education campaigns could help close this gap and foster preventive behaviors [47].

Health care inequities and structural barriers were underlined. Individuals with formal long-term illness coverage were more likely to have a confirmed long COVID diagnosis, suggesting that care access influences diagnostic likelihood. This may reflect better access to medical specialists, administrative support, or continuity of care within chronic disease networks [52,53]. Conversely, many suspected cases may remain undiagnosed due to logistical, economic, or informational barriers [54,55].

Diagnosing long COVID remains challenging, with many cases potentially undetected due to health care inequities, making SARS-CoV-2 monitoring essential. Between May 26, 2025, and June 1, 2025, WHO recorded 60,216 SARS-CoV-2 tests from 80 countries, with 4.1% (2471 samples) positive [34]. While worldwide COVID-19 activity was low, localized increases appeared in many countries. These data underscore ongoing risks of post-COVID conditions including in France and the need for proactive workplace prevention strategies, particularly within the public sector, where long COVID can disrupt essential services. Recommended interventions include occupational health screening for post-COVID symptoms, tailored reintegration plans for affected employees, awareness campaigns, flexible work policies, and accommodations for cognitive or physical impairments and mental health support.

In France, implementing these interventions would align with existing recommendations from the HAS and the *Conseil scientifique* COVID-19 (state scientific council), which emphasize early identification and personalized return-to-work pathways for post-COVID employees [56,57]. Occupational health services within the public sector could play a central role in this process by reinforcing health surveillance, facilitating multidisciplinary coordination, and adapting workplace accommodations. The WHO also recommends integrating long COVID into occupational health monitoring and risk assessment systems to improve prevention and equity [44]. Such structured reintegration and follow-up programs would strengthen the resilience of public institutions while supporting long-term workforce health. Such strategies protect both individual health and institutional productivity and resilience. OECD estimates long COVID costs between $864 billion and $1.04 trillion across high-income countries [58], mainly from health care expenditures and indirect productivity losses.

### Limitations

This study had several limitations. First, the cross-sectional design limits causal interpretations, as associations cannot be interpreted as temporal or causal associations. Second, outcomes relied on self-reported data, which may introduce bias at several levels: recall bias (participants may not accurately remember or report their COVID-19 history or symptom duration), misclassification bias (symptoms could be wrongly attributed to or excluded from long COVID), and reporting bias (participants may over- or under-report sensitive information such as vaccination status or comorbidities). Third, no validated quality of life instruments were used, limiting conclusions regarding functional and psychosocial impact. Fourth, the sampling strategy may also affect representativeness and generalizability: participants were recruited through convenience sampling of UROPS members and voluntary participation, which introduces self-selection bias. Individuals with a stronger interest in COVID-19 or more health concerns may have been more likely to participate, leading to overrepresentation of certain profiles and underrepresentation of others. In addition, the overall response rate was low (6.12%), which may further accentuate selection bias. Moreover, generalizability is limited: although seemingly applicable to all 5.67 million French public servants, the study focused only on 1.46 million state agents, excluding 1.06 million in national education [28]. Fifth, the questionnaire did not assess whether vaccination occurred before or after SARS-CoV-2 infection, preventing interpretation of the temporal sequence and limiting conclusions. Finally, psychometric properties other than the Content Validity Index, such as internal consistency or test-retest reliability, were not assessed, as the knowledge items measured factual information rather than a latent construct, and knowledge levels may legitimately change over time with exposure to new information. In addition, the cognitive debriefing involved only six individuals; although this number is consistent with standard practice, it may still limit robustness. The knowledge score was validated internally but not benchmarked against external instruments or tested in known-groups comparisons, which may limit external validity. Future studies should consider using probability sampling strategies or weighting procedures to improve representativeness and strengthen external validity.

### Conclusions

In a context of widespread SARS-CoV-2 infection, most French civil servants had experienced a past infection and a smaller but substantial proportion reported long COVID, either diagnosed or suspected. Disparities in knowledge about long COVID, attitudes, and behaviors toward COVID-19 vaccination were observed. Importantly, vaccination was found to be associated with a lower odds ratio of developing long COVID. These findings have implications for workplace prevention suggesting the value of health education, post-COVID screening for persistent symptoms, tailored reintegration plans, vaccination promotion, and mental health support. Enhancing disease literacy and occupational health programs could help strengthen the resilience of essential public services.

## Supplementary material

10.2196/83323 Multimedia Appendix 1 Final validated study questionnaire.

10.2196/83323 Multimedia Appendix 2 Classification of participants by COVID-19 and long COVID status.

10.2196/83323 Multimedia Appendix 3 Sociodemographic and health characteristics of the respondents.

10.2196/83323 Multimedia Appendix 4 Analysis of long COVID knowledge.

10.2196/83323 Checklist 1 CROSS checklist.

## Abbreviations

CROSS: Checklist for Reporting of Survey Studies
HAS: Haute Autorité de la Santé
I-CVI: Item-level Content Validity Index
KAB: Knowledge, Attitudes, and Behaviors
OECD: Organization for Economic Co-operation and Development
OR: odds ratio
S-CVI/Ave: Scale-level Content Validity Index
UROPS: Union Prévention Santé pour la Fonction publique
WHO: World Health Organization
