# Supplementary material for: Prevalence, Risk Factors, Disease-Related Knowledge, and Vaccination Attitudes and Behaviors for Long COVID Among French Civil Servants: Cross-Sectional Survey
Source: JMIR Public Health Surveill. 2025 Dec 5;11:e83323. doi: 10.2196/83323 (PMC12680290; doi:10.2196/83323)
Supplement: Multimedia Appendix 1 [file publichealth-v11-e83323-s001.docx]

**Multimedia Appendix 1.** Final validated study questionnaire

**Socio-demographic characteristics**

Q1. Are you:

1. Male
2. Female

Q2. How old are you?

1. 18-29 years old
2. 30-39 years old
3. 40-49 years old
4. 50-59 years old
5. 60-69 years old
6. 70-79
7. 80 or older

Q3. What is your marital status?

1. Single
2. In a relationship
3. Divorced
4. Widowed

Q4. Do you have children?

1. Yes
2. No

If you have children (Q4=1)

Q5. Do you live with your children?

1. Yes
2. No

Q6. What is your current employment status?

1. Working
2. On parental leave
3. In school, training, or internship
4. Retired
5. Disabled
6. On sick leave for more than 3 months

Q7. What is (or was) your last statutory category?

1. Category A+ permanent employee
2. Category A permanent employee
3. Category B permanent employee
4. Category C permanent employee
5. Contract employee
6. Other

Q8. What is the highest level of education you have attained?

1. No diploma
2. Middle school diploma
3. CAP or BEP
4. General or vocational high school diploma
5. Two-year post-secondary diploma (DUT, BTS, DEUG, health or social services training school, etc.)
6. Secondary degree (bachelor's degree, master's degree, master's 1, etc.)
7. Tertiary degree (master's 2, DEA, DESS, doctorate or graduate school degree, etc.)

Q9. Which region do you live in?

1. Auvergne-Rhône-Alpes
2. Bourgogne-Franche-Comté
3. Brittany
4. Centre-Val-de-Loire
5. Corsica
6. Grand Est
7. Hauts-de-France
8. Île-de-France
9. Normandy
10. Nouvelle Aquitaine
11. Occitanie
12. Pays de la Loire
13. Provence-Alpes-Côte-d'Azur
14. Overseas

Q10. How many inhabitants are there in the municipality where you live?

1. Rural area: fewer than 2,000 inhabitants
2. Small urban area: 2,000 to 20,000 inhabitants
3. Medium-sized urban area: 20,000 to 100,000 inhabitants
4. Large urban area: 100,000 inhabitants or more
5. Paris metropolitan area

Q11. Which government department do you belong to?

1. Ministry of the Economy, Finance, and Industrial and Digital Sovereignty
2. Ministry of the Interior and Overseas Territories
3. Ministry for Europe and Foreign Affairs
4. Ministry of Justice
5. Ministry of the Armed Forces
6. Ministry of Labor, Full Employment, and Integration
7. Ministry of Agriculture and Food Sovereignty
8. Ministry of Health and Prevention
9. Ministry of Solidarity, Autonomy, and People with Disabilities
10. Ministry of Transformation and Public Service
11. Other public services

Q12. What type of job do you do?

1. Multiple answers possible except “don't know”
2. Sedentary (administrative, office-based)
3. Active (mobile, field-based, often traveling)
4. In contact with the public (reception, etc.)
5. Don't know

**Health status**

Q13. Do you have a chronic illness (diabetes, asthma, etc.), a disability, or a health problem that has been concerning you for at least six months and requires regular care or treatment?

1. Yes
2. No
3. I don't know

*If you have a chronic illness, disability, or health problem (Q13=1)*

Q14. What type(s) of condition(s) do you have?

1. Random rotation of items except for “Other,” which is always last.
2. Multiple answers possible.
3. Respiratory disease (asthma, chronic bronchitis, etc.)
4. Cardiovascular disease (heart disease, artery disease, vein disease, stroke, etc.)
5. Diabetes
6. Treated or monitored high blood pressure
7. Treated or monitored high cholesterol
8. Kidney problems (kidney failure, nephritis, kidney stones, etc.)
9. Neurological disease (multiple sclerosis, cognitive disorders/dementia, etc.)
10. Inflammatory bowel disease (Crohn's disease, ulcerative colitis, IBD, etc.)
11. Tumors (cancers, malignant tumors, lymphoma, leukemia, etc.)
12. Mental health (depression, anxiety disorders, sleep disorders, etc.)
13. Musculoskeletal disorders and/or functional limitations and/or pain (back, upper limbs, lower limbs, etc.)
14. Migraine
15. Rare diseases
16. Other, please specify: ……………………………

Q15. Are you currently covered 100% by health insurance for a long-term condition?

1. Yes
2. No
3. Don't know

Q16. Have you ever been treated or are you currently being treated for a chronic immunological disease (polyarthritis, lupus, hives, etc.)?

1. Yes
2. No
3. Don't know

Q17. Have you ever been treated or are you currently being treated for cancer?

1. Yes
2. No
3. Prefer not to answer

**Variable section – Long COVID**

Q18. Have you ever had COVID-19?

1. Yes, once
2. Yes, several times
3. No
4. Don't know

*If you have already had COVID-19 (Q18=1 or 2)*

Q19. When did you last have COVID-19?

1. Less than 1 month ago
2. Between 1 month and less than 3 months ago
3. Between 3 months and less than 6 months ago
4. 6 months or more ago
5. Don't know

*If you have already had COVID-19 (Q18=1 or 2)*

Q20. Were you vaccinated the last time you contracted COVID-19 or in the previous year?

1. Yes
2. No
3. Don't know

*If you have already contracted COVID-19 (Q18=1 or 2)*

Q21. Have you been hospitalized for COVID-19?

1. Multiple answers possible except no
2. Yes, in intensive care
3. Yes, in follow-up care and rehabilitation
4. Yes, in another department
5. No

*If you have already contracted COVID-19 (Q18=1 or 2)*

Q22. Did you have phlebitis, pulmonary embolism, or other thrombosis during your COVID-19 infection?

1. Yes
2. No
3. Don't know

*If you have already contracted COVID-19 (Q18=1 or 2)*

Q23. Have you been diagnosed with long COVID-19?

1. Yes
2. No, but I think I have it or have had it
3. No, and I don't think I've had it
4. Don't know

*If you think you have had long COVID-19 without being diagnosed (Q23=2)*

Q24. Have you consulted a doctor about this possible long COVID-19?

1. Yes
2. No
3. Don't know

*If you have not been diagnosed with long COVID-19 and think you have had long COVID* *(Q23=2)*

The following questions are about the symptoms you may have experienced during your COVID-19 infection.

During the different phases of your COVID-19 infection, which of these symptoms did you experience?

|  | Q25. During the acute phase of the disease | Q26. For 4 weeks or more | Q27. Currently |
| --- | --- | --- | --- |
|  | If you have not been diagnosed with long Covid-19 and do not think you have had long Covid or nsp (Q22=3,4) | If you experienced at least one symptom (Q24=1 to 15) | If you experienced at least one symptom (Q24=1 to 15) |
| 1. Fatigue |  |  |  |
| 1. Concentration or memory problems |  |  |  |
| 1. Headaches |  |  |  |
| 1. Fever |  |  |  |
| 1. Severe muscle pain |  |  |  |
| 1. Chest pain, tightness, or palpitations |  |  |  |
| 1. Breathing difficulties, coughing |  |  |  |
| 1. High blood pressure |  |  |  |
| 1. Smell disorders |  |  |  |
| 1. Taste disorders |  |  |  |
| 1. Pain when swallowing |  |  |  |
| 1. Joint pain |  |  |  |
| 1. Abdominal pain |  |  |  |
| 1. Intestinal transit disorders |  |  |  |
| 1. Skin disorders |  |  |  |
| 1. I have not had any of these symptoms |  |  |  |

**Knowledge**

Q28. Does COVID-19 is a viral disease?

1. Yes
2. No
3. Don't know

Q29. Does long COVID-19 is a viral disease?

1. Yes
2. No
3. Don't know

Q30. Does long COVID-19 is an immunological disease?

1. Yes
2. No
3. Don't know

Q31. Does long COVID-19 is a contagious disease?

1. Yes
2. No
3. Don't know

Q32. In your opinion, long COVID-19 is a disease whose symptoms...

*Multiple answers possible except “Don't know”*

1. Persist beyond 4 weeks
2. Become more numerous over time
3. Are not related to my usual illnesses
4. Are not found in any other disease
5. Don't know

Q33. In your opinion, COVID-19 is diagnosed by...

*Multiple answers possible except “Don't know”*

1. PCR
2. Antigen test
3. Analysis of symptoms
4. Chest scan
5. Don't know

Q34. In your opinion, long COVID-19 may be related to...

*Multiple answers possible except “Don't know”*

1. Inadequate treatment during the early symptoms of COVID-19
2. Taking certain medications during the early symptoms of COVID-19
3. Initial state of health
4. Don't know

**Attitudes and behavior regarding vaccination**

*If has already contracted COVID-19 (Q17=1 or 2)*

Q35. Do you intend to get vaccinated against COVID-19?

1. Yes
2. No, I am already vaccinated
3. No, and I am not vaccinated

*If does not intend to get vaccinated and is not vaccinated (Q34=3)*

Q36. For what reason(s) do you not intend to get vaccinated against COVID-19?

*Maximum of 5 answers*

1. Not effective in preventing me from getting sick
2. Don't trust vaccination, risk of side effects
3. Don't trust the vaccine's effectiveness
4. I am immune, I have already had COVID-19
5. It doesn't matter if I get infected with this virus, the disease is not serious
6. Prefer other means of prevention, such as protective measures, wearing a mask, or physical distancing
7. I don't like injections
8. Against vaccination in general
9. Too complicated to get vaccinated
10. There is little or no COVID-19 currently
11. Other reason

*If you intend to get vaccinated (Q34=1)*

Q36. For what reason(s) do you intend to get vaccinated against COVID-19?

*Multiple answers possible.*

1. The COVID-19 vaccine has already protected me from a more severe form of the disease
2. I need to protect myself from new variants
3. I need to boost my immunity
4. I need to protect my loved ones
5. I am following the advice of my doctor/pharmacist...
6. I am following the recommendations of the health authorities
7. Other reason
