# Supplementary material for: Prevalence, Risk Factors, Disease-Related Knowledge, and Vaccination Attitudes and Behaviors for Long COVID Among French Civil Servants: Cross-Sectional Survey
Source: JMIR Public Health Surveill. 2025 Dec 5;11:e83323. doi: 10.2196/83323 (PMC12680290; doi:10.2196/83323)
Supplement: Multimedia Appendix 2 [file publichealth-v11-e83323-s002.docx]

**Multimedia Appendix 2.** Classification of Participants by COVID-19 and Long COVID Status

The definition of Long COVID used in this study was based on HAS recommendations, describing it as the persistence of one or more symptoms beyond four weeks after the acute phase of infection, without another diagnosis explaining them [29]. Participants were categorized into four mutually exclusive groups based on self-reported infection history and symptomatology:

- No COVID: participants who reported never having had a SARS-CoV-2 infection (Q18 answer 3).
- COVID without long COVID: participants who reported a previous SARS-CoV-2 infection but no persistent symptoms compatible with long COVID (Q18 answer 1 or 2 and Q23 answer 3).
- Diagnosed long COVID: participants who reported a formal medical diagnosis of long COVID following a confirmed SARS-CoV-2 infection (Q18 answer 1 or 2 and Q23 answer 1).
- Suspected long COVID: participants who reported persistent symptoms consistent with long COVID but without a formal medical diagnosis (Q18 answer 1 or 2 and Q23 answer 2 and Q26 at least 1symptom selected).

These groups were subsequently used for descriptive comparisons and KAB analyses.
