# Supplementary material for: Prevalence, Risk Factors, Disease-Related Knowledge, and Vaccination Attitudes and Behaviors for Long COVID Among French Civil Servants: Cross-Sectional Survey
Source: JMIR Public Health Surveill. 2025 Dec 5;11:e83323. doi: 10.2196/83323 (PMC12680290; doi:10.2196/83323)
Supplement: Multimedia Appendix 3 [file publichealth-v11-e83323-s003.docx]

**Multimedia Appendix 3.** Socio-demographic and health characteristics of the respondents.

The results are expressed as n (%). n: number of respondents, %: percentage.

| **Variable** | **All**  **n (%)** | **No Covid**  **n (%)** | **Covid without long Covid**  **n (%)** | **Diagnosed long Covid**  **n (%)** | **Suspected long Covid**  **n (%)** |  |
| --- | --- | --- | --- | --- | --- | --- |
| **Having children** |  |  |  |  |  |  |
| Yes | 3195 (80.6%) | 1088 (79.0%) | 1702 (81.6%) | 43 (70.5%) | 197 (81.7%) |  |
| No | 767 (19.4%) | 290 (21.0%) | 385 (18.4%) | 18 (29.5%) | 44 (18.3%) |  |
| **Children living in the household** |  |  |  |  |  |  |
| Yes | 1040 (26.2%) | 281 (20.4%) | 604 (28.9%) | 22 (36.1%) | 70 (29.0%) |  |
| No | 2155 (54.4%) | 807 (58.6%) | 1098 (52.6%) | 21 (34.4%) | 127 (52.7%) |  |
| **Region of residence** |  |  |  |  |  |  |
| Auvergne-Rhône-Alpes | 429 (10.8%) | 140 (10.2%) | 231 (11.1%) | 2 (3.3%) | 28 (11.6%) |  |
| Bourgogne-Franche-Comté | 186 (4.7%) | 57 (4.1%) | 100 (4.8%) | 1 (1.6%) | 10 (4.1%) |  |
| Bretagne | 244 (6.2%) | 95 (6.9%) | 123 (5.9%) | 1 (1.6%) | 17 (7.1%) |  |
| Centre-Val-de-Loire | 235 (5.9%) | 88 (6.4%) | 114 (5.5%) | 6 (9.8%) | 16 (6.6%) |  |
| Corse | 26 (0.7%) | 9 (0.7%) | 14 (0.7%) | 0 (0.0%) | 2 (0.8%) |  |
| Grand Est | 319 (8.1%) | 107 (7.8%) | 176 (8.4%) | 1 (1.6%) | 20 (8.3%) |  |
| Hauts-de-France | 280 (7.1%) | 107 (7.8%) | 135 (6.5%) | 7 (11.5%) | 20 (8.3%) |  |
| Île-de-France | 546 (13.8%) | 173 (12.6%) | 317 (15.2%) | 11 (18.0%) | 23 (9.5%) |  |
| Normandie | 164 (4.1%) | 68 (4.9%) | 81 (3.9%) | 2 (3.3%) | 8 (3.3%) |  |
| Nouvelle Aquitaine | 469 (11.8%) | 156 (11.3%) | 247 (11.8%) | 6 (9.8%) | 30 (12.4%) |  |
| Occitanie | 427 (10.8%) | 146 (10.6%) | 224 (10.7%) | 7 (11.5%) | 35 (14.5%) |  |
| Pays de la Loire | 203 (5.1%) | 75 (5.4%) | 106 (5.1%) | 3 (4.9%) | 11 (4.6%) |  |
| Provence-Alpes-Côte-d'Azur | 241 (6.1%) | 75 (5.4%) | 137 (6.6%) | 6 (9.8%) | 15 (6.2%) |  |
| Outremer | 193 (4.9%) | 82 (6.0%) | 82 (3.9%) | 8 (13.1%) | 6 (2.5%) |  |
| **Administration** |  |  |  |  |  |  |
| Ministry of the Economy, Finance and Industrial and Digital Sovereignty | 1502 (37.9%) | 539 (39.1%) | 804 (38.5%) | 21 (34.4%) | 74 (30.7%) |  |
| Ministry of the Interior and Overseas France | 402 (10.1%) | 143 (10.4%) | 199 (9.5%) | 9 (14.8%) | 28 (11.6%) |  |
| Ministry of Europe and Foreign Affairs | 3 (0.1%) | 1 (0.1%) | 1 (0.0%) | 0 (0.0%) | 1 (0.4%) |  |
| Ministry of Justice | 384 (9.7%) | 124 (9.0%) | 189 (9.1%) | 8 (13.1%) | 36 (14.9%) |  |
| Ministry of the Armed Forces | 432 (10.9%) | 166 (12.0%) | 213 (10.2%) | 3 (4.9%) | 23 (9.5%) |  |
| Ministry of Labor, Employment and Integration | 98 (2.5%) | 33 (2.4%) | 50 (2.4%) | 1 (1.6%) | 8 (3.3%) |  |
| Ministry of Agriculture and Food Sovereignty | 299 (7.5%) | 97 (7.0%) | 169 (8.1%) | 2 (3.3%) | 18 (7.5%) |  |
| Ministry of Health and Prevention | 194 (4.9%) | 75 (5.4%) | 96 (4.6%) | 5 (8.2%) | 11 (4.6%) |  |
| Ministry of Solidarity, Autonomy and the Disabled | 50 (1.3%) | 20 (1.5%) | 27 (1.3%) | 1 (1.6%) | 1 (0.4%) |  |
| Ministry of Public Transformation and Civil Service | 53 (1.3%) | 20 (1.5%) | 24 (1.1%) | 1 (1.6%) | 2 (0.8%) |  |
| Other public services | 545 (13.8%) | 160 (11.6%) | 315 (15.1%) | 10 (16.4%) | 39 (16.2%) |  |
| **Type of job** |  |  |  |  |  |  |
| Sedentary (administrative, office-based) | 2575 (65.0%) | 869 (63.1%) | 1410 (67.6%) | 35 (57.4%) | 139 (57.7%) |  |
| Active (mobile, in the field, often on the move) | 904 (22.8%) | 330 (23.9%) | 465 (22.3%) | 17 (27.9%) | 58 (24.1%) |  |
| In contact with the public (reception, etc.) | 319 (8.1%) | 107 (7.8%) | 142 (6.8%) | 6 (9.8%) | 35 (14.5%) |  |
| Don't know | 164 (4.1%) | 72 (5.2%) | 70 (3.4%) | 3 (4.9%) | 9 (3.7%) |  |
| **Chronic disease or disability or health problem** |  |  |  |  |  |  |
| Yes | 1840 (46.4%) | 637 (46.2%) | 958 (45.9%) | 39 (63.9%) | 107 (44.4%) |  |
| No | 1976 (49.9%) | 690 (50.1%) | 1069 (51.2%) | 19 (31.1%) | 120 (49.8%) |  |
| Don’t want to answer | 0 (0.0%) | 0 (0.0%) | 0 (0.0%) | 0 (0.0%) | 0 (0.0%) |  |
| **Type of chronic disease or disability or health problem^1^** |  |  |  |  |  |  |
| Respiratory disease | 232 (5.9%) | 70 (5.1%) | 119 (5.7%) | 11 (18.0%) | 13 (5.4%) |  |
| Cardio-vascular disease | 339 (8.6%) | 128 (9.3%) | 174 (8.3%) | 6 (9.8%) | 13 (5.4%) |  |
| Diabetes | 265 (6.7%) | 112 (8.1%) | 119 (5.7%) | 6 (9.8%) | 14 (5.8%) |  |
| Hypertension treated or monitored | 603 (15.2%) | 233 (16.9%) | 300 (14.4%) | 15 (24.6%) | 24 (10.0%) |  |
| Hypercholesterolemia treated or monitored | 229 (5.8%) | 92 (6.7%) | 113 (5.4%) | 1 (1.6%) | 11 (4.6%) |  |
| Kidney problems | 76 (1.9%) | 37 (2.7%) | 33 (1.6%) | 0 (0.0%) | 1 (0.4%) |  |
| Neurological disease | 82 (2.1%) | 33 (2.4%) | 37 (1.8%) | 3 (4.9%) | 2 (0.8%) |  |
| Inflammatory disease of the digestive tract | 66 (1.7%) | 17 (1.2%) | 36 (1.7%) | 1 (1.6%) | 9 (3.7%) |  |
| Tumors | 208 (5.2%) | 71 (5.2%) | 112 (5.4%) | 3 (4.9%) | 11 (4.6%) |  |
| Mental health (depression, anxiety disorders, sleep disorders, etc.) | 229 (5.8%) | 65 (4.7%) | 124 (5.9%) | 9 (14.8%) | 17 (7.1%) |  |
| Musculoskeletal disorders and/or functional limitations and/or pain (back, upper limbs, lower limbs...) | 415 (10.5%) | 129 (9.4%) | 227 (10.9%) | 10 (16.4%) | 29 (12.0%) |  |
| Migraine | 95 (2.4%) | 18 (1.3%) | 51 (2.4%) | 5 (8.2%) | 11 (4.6%) |  |
| Rare diseases | 78 (2.0%) | 28 (2.0%) | 36 (1.7%) | 6 (9.8%) | 2 (0.8%) |  |
| Other | 39 (1.0%) | 14 (1.0%) | 21 (1.0%) | 0 (0.0%) | 3 (1.2%) |  |
| **Reimbursed at 100% by the Assurance Maladie for a long-term illness** |  |  |  |  |  |  |
| Yes | 1175 (29.7%) | 461 (33.5%) | 553 (26.5%) | 28 (45.9%) | 63 (26.1%) |  |
| No | 2706 (68.3%) | 892 (64.7%) | 1493 (71.5%) | 31 (50.8%) | 175 (72.6%) |  |
| Don’t know | 0 (0.0%) | 0 (0.0%) | 0 (0.0%) | 0 (0.0%) | 0 (0.0%) |  |
| **Already treated or currently being treated for a chronic immunological disease (polyarthritis, lupus, urticaria...)** |  |  |  |  |  |  |
| Yes | 249 (6.3%) | 82 (6.0%) | 129 (6.2%) | 6 (9.8%) | 18 (7.5%) |  |
| No | 3650 (92.1%) | 1271 (92.2%) | 1935 (92.7%) | 55 (90.2%) | 215 (89.2%) |  |
| Don’t want to answer | 0 (0.0%) | 0 (0.0%) | 0 (0.0%) | 0 (0.0%) | 0 (0.0%) |  |
| **Already treated or currently being treated for a cancer** |  |  |  |  |  |  |
| Yes | 483 (12.2%) | 185 (13.4%) | 241 (11.5%) | 8 (13.1%) | 24 (10.0%) |  |
| No | 3448 (87.0%) | 1174 (85.2%) | 1841 (88.2%) | 53 (86.9%) | 213 (88.4%) |  |
| Don’t want to answer | 31 (0.8%) | 19 (1.4%) | 5 (0.2%) | 0 (0.0%) | 4 (1.7%) |  |
