# Supplementary material for: Prevalence, Risk Factors, Disease-Related Knowledge, and Vaccination Attitudes and Behaviors for Long COVID Among French Civil Servants: Cross-Sectional Survey
Source: JMIR Public Health Surveill. 2025 Dec 5;11:e83323. doi: 10.2196/83323 (PMC12680290; doi:10.2196/83323)
Supplement: Multimedia Appendix 4 [file publichealth-v11-e83323-s004.docx]

**Multimedia Appendix 4.** Analysis of long COVID knowledge

| Group | **Low**  n (%[95% CI]) | **Medium**  n (%[95% CI]) | **High**  n (%[95% CI]) |
| --- | --- | --- | --- |
| Total population (N = 3962) | 758 (19.1%[17.9–20.4]) | 2565 (64.7%[63.2–66.2]) | 639 (16.1%[15.0–17.3]) |
| Diagnosed long COVID (N = 61) | 10 (16.4%[9.2–27.6]) | 38 (62.3%[49.7–73.4]) | 13 (21.3%[12.9–33.1]) |
| Suspected long COVID (N = 241) | 39 (16.2%[12.1–21.4]) | 163 (67.6%[61.5–73.2]) | 39 (16.2%[12.1–21.4]) |
| COVID without long COVID (N = 2087) | 302 (14.5%[13.0–16.0]) | 1387 (66.5%; [64.4–68.5]) | 398 (19.1%[17.4–20.8]) |
| Non COVID (N = 1091) | 257 (23.6%[21.1–26.2]) | 699 (64.1%[61.2–66.9]) | 135 (12.4%[10.6–14.5]) |

**ANOVA test (comparison of mean K-scores between groups):**

- F-statistic = 24.31
- p-value < 0.00001
  **Significant result:** The mean K-scores differ significantly across groups.

**Chi-square test (association between group and knowledge level – categorized K-score):**

- Chi²-statistic = 54.92
- p-value < 0.00001
- Degrees of freedom = 6
  **Significant result:** The distribution of knowledge levels (low, medium, high) varies significantly across groups.

**Post-hoc Chi-square pairwise comparisons:**

- Diagnosed long Covid vs Non-Covid: mean difference = –0.72, p = 0.0021
- Suspected long Covid vs Non-Covid: mean difference = –0.35, p = 0.0081
- Covid without long Covid vs Non-Covid: mean difference = –0.47, p < 0.001
